# Biological Role of Vitamin K—With Particular Emphasis on Cardiovascular and Renal Aspects

**DOI:** 10.3390/nu14020262

**Published:** 2022-01-08

**Authors:** Anna Stępień, Małgorzata Koziarska-Rościszewska, Jacek Rysz, Mariusz Stępień

**Affiliations:** 1 Department of Nephrology, Hypertension and Family Medicine, Medical University of Lodz, Żeromski St. 113, 90-549 Lodz, Poland; anna.stepien@umed.lodz.pl (A.S.); malgorzata.koziarska-rosciszewska@umed.lodz.pl (M.K.-R.); jacek.rysz@umed.lodz.pl (J.R.); 2 Department of Propaedeutics of Internal Medicine and Social Pharmacology, Medical University of Lodz, Żeromski St. 113, 90-549 Lodz, Poland

**Keywords:** vitamin K, chronic kidney disease, vascular calcification, hemodialysis, vitamin K supplementation, calciphylaxis, anticoagulants

## Abstract

Vitamin K (VK) plays many important functions in the body. The most important of them include the contribution in calcium homeostasis and anticoagulation. Vascular calcification (VC) is one of the most important mechanisms of renal pathology. The most potent inhibitor of this process—matrix Gla protein (MGP) is VK-dependent. Chronic kidney disease (CKD) patients, both non-dialysed and hemodialysed, often have VK deficiency. Elevated uncarboxylated matrix Gla protein (ucMGP) levels indirectly reflected VK deficiency and are associated with a higher risk of cardiovascular events in these patients. It has been suggested that VK intake may reduce the VC and related cardiovascular risk. Vitamin K intake has been suggested to reduce VC and the associated cardiovascular risk. The role and possibility of VK supplementation as well as the impact of anticoagulation therapy on VK deficiency in CKD patients is discussed.

## 1. Introduction

Vitamin K (VK) is a fat-soluble vitamin, less popular compared to others, but performs many very important functions in the body [1,2]. Vitamin K was discovered by Henrik Dam in 1935 [3]. In nature there occur two types of vitamin K—vitamin K_1_ (phylloquinone, PK, VK1) and vitamin K_2_ (menaquinone, VK2, MK-n, where n means isoprene units count and MK comprises 15 types) [1,2,3,4,5,6,7]. Vitamin K3 (menadione, VK3), a synthetic form of vitamin K, is a provitamin and is characterized by the absence of a side chain [2]. Vitamin K_1_ is mainly found in green leafy vegetables, especially spinach, broccoli, kale, olive oil, as well as soyabean oil, whereas vitamin K_2_ is present in fish, chicken, milk, liver, cheese, butter, egg yolks, fermented soyabeans (natto) and vegetables, however only in small amounts [1,2,3,5,6,7,8,9,10]. Although intestinal bacterial flora in mammalians can produce vitamin K_2_, these amounts are insufficient to cover the demand [1,2,3].

VK2, in contrast to VK1, is not stored in the liver but in other tissues, especially in bones and blood vessels [10]. VK2 has a longer half-life (days) than VK1 (hours) [4]. VK2 activates extrahepatic vitamin K-dependent proteins (VKDPs) to a greater extent than VK1 [4]. After intestinal absorption VK is solubilized by bile salt and pancreatic juice and then joins with chylomicrons into the lymphatic system [7]. Therefore, lipids, especially triglycerides may interfere with VK measurements. VK is recycled in several redox reactions. Menaquinone-4, which is the form of vitamin K_2_, can be produced as a result of the endogenous conversion of phylloquinone [6].

## 2. Biological Role of VK

**Figure 1:**
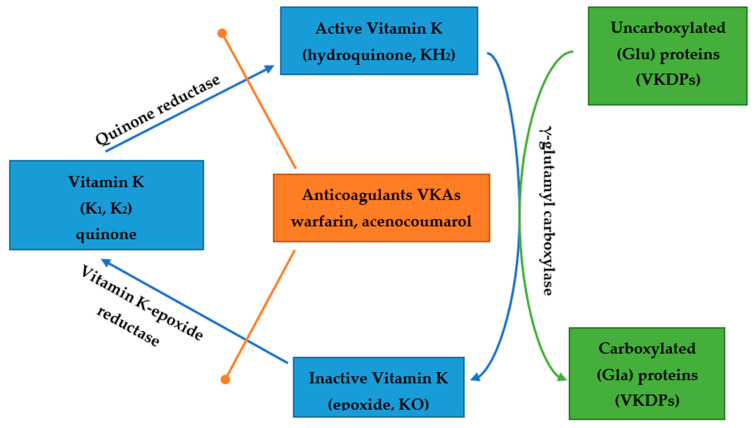
The vitamin K cycle and action. VKAs—vitamin K antagonists, VKDPs—vitamin K-dependent proteins, Glu-glutamate, Gla-γ-carboxyglutamate, KH_2_-vitamin K hydroquinone, KO-vitamin K epoxide.

Vitamin K is necessary for appropriate activity of VKDPs [3,9,10,11,12]. VK1 and VK2 are cofactors for the enzyme γ-glutamyl carboxylase. This enzyme catalyzes the attachment of carboxyl groups to Glu residues in proteins, whose carboxylation is vitamin K dependent [3,12,13]. This group consists of at least 18 proteins which include several proteins involved in blood clotting (protein C, S, M, Z, factors VII, IX, X and prothrombin), Gas6 (Growth Arrest-Specific 6 Protein), bone Gla protein (BGP or osteocalcin OC), matrix Gla protein (MGP), periostin and GRP (Gla Rich Protein) [6,7,9,10,11,12,13]. The most important VKDPs are prothrombin, MGP and OC [6,7,10,11]. These proteins are important for coagulation, protection against vascular calcification (VC) and for bone mineralization. One of the largest VKDPs is Gas 6 weighting 75 kDa. Its plasma levels in healthy humans range from approximately 2.5 to 18.8 μg/L [3]. This protein is highly homologous to the protein S and contains an N-terminal Gla domain after vitamin K carboxylation [3]. Gas widely occurs in heart, brain, kidney, lung and other tissues except liver and is considered to be the endogenous ligand for the TAM. TAM stands for the following three receptors: Tyro3, Axl and Mer [3]. Axl is the receptor with the greatest affinity to Gas 6 [3]. The vitamin K-dependent carboxylation is of greatest importance for the interaction between Gas 6 and the TAM receptor [3]. Vitamin K’s cycle and actions are presented in Figure 1.

All these proteins play very important roles in the human organism. Thus, carbo-xylated Gas 6 and GRP take part in the protection of blood vessels against calcification, Gas 6 protects the kidneys from acute damage and plays a role in chronic kidney disease (CKD), GRP takes part in bone homeostasis and in delaying the development of osteoarthritis, whereas periostin takes part in all phases of fracture healing, and in early myocardial regeneration after myocardial infarction. Moreover, periostin is also involved in the development of airway remodeling of asthma, as well as in both cardiac and idiopathic pulmonary fibrosis [3].

The most known function associated with vitamin K is its participation in the blood coagulation process. Vitamin K is needed for the synthesis and action of coagulation factors, such as factor II, VII, IX and X (the so-called prothrombin complex) and both proteins C and S [1,10]. This phenomenon is used in the anticoagulant treatment with hydroxycoumarin derivatives such as acenocoumarol or warfarin, which are inhibitors of vitamin K synthesis [1].

It was reported that vitamin K might also inhibit the growth of some cancer cells in humans e.g., hepatocellular carcinoma in cirrhotic patients [1,10].

Vitamin K is an important element of the synthesis and action of osteocalcin, a bone-forming protein [1,10,11]. Vitamin K is associated with the carboxylation of bone-related proteins, which regulate genetic transcription of osteoblastic markers as well as bone reabsorption [9,10,11]. It has been recently reported that VK2 controlled osteoblastogenesis and osteoclastogenesis by the NF-κB signal transduction pathway [2]. Osteocalcin deficiency leads to osteoporosis, which greatly increases the risk of bone fractures [1,10]. Low serum concentrations of VK1, high levels of undercarboxylated osteocalcin (ucOC), a marker of VK deficiency, and low VK1 an VK2 contents in the diet are associated with a higher risk of bone fractures and lower bone mineral density (BMD) [10]. Elevated serum ucOC is often found in patients with CKD with hyperparathyroidism [4,11]. However, it does not generally suggest VK deficiency [4].

Vitamin K is important for the regulation of the glycemic status by reducing the risk of developing diabetes mellitus and improving insulin sensitivity [1,14]. It was shown that vitamin K had an impact on pancreatic β-cell proliferation and on adiponectin synthesis [1]. Menaquinone-4 (MK-4) a homolog of VK2, enhances glucose-stimulated insulin secretion in isolated mouse islets and INS-1 rat insulinoma cells [15]. Vitamin K is also a cofactor for microsomal-glutamyl carboxylase which is very important for the posttranslational carboxylation of glutamate to γ-carboxyglutamate (Gla) residues of VKDPs, among them, OC [10,14].

Vitamin K consumption and a long-term status of vitamin K are expressed by a high percentage of ucOC, which plays a role of an endocrine hormone, which impacts glucose metabolism, energy metabolism and fertility [4,14]. The data obtained from animal studies indicated that ucOC improved insulin sensitivity and enhanced β-cell functions via the stimulation of cyclin D1 and insulin expression in β cells and adiponectin expression in adipocytes [14]. On the other hand, results of clinical trials suggest that the protective effect of VK on the progression of insulin resistance might be related with decreasing levels of ucOC [14]. Other clinical studies also showed the important role of OC in the regulation of glucose metabolism via increased insulin secretion and expression of adiponectin [14]. It has been suggested that VK may impact insulin response and glycemic status by inhibiting inflammation [14]. Results of some studies showed that VK suppressed IL-6 synthesis in lipopolysaccharide-induced inflammation models, and, what is more, high VK1 intake and plasma levels were correlated with lowered concentrations of inflammatory markers such as TNF-α and IL-6 [14].

Thus, VK performs many functions in the body and some of which require more extensive studies to determine their practical significance. Nevertheless, the best-known functions of this vitamin with the greatest clinical significance include participation in the process of blood clotting, blood vessel calcification and bone metabolism.

## 3. Vitamin K, Cardiovascular Risk and Vascular Calcification

VK deficiency is regarded as an independent predictor of cardiovascular disease (CVD) risk and supplementation of vitamin K may slow this process and prevent atherosclerosis, as well as CVD and stroke [2].

Vascular calcification is a dynamic process with the participation of calcification promoters and inhibitors and, at present, no particular therapy is known [2,11]. The mechanism of this process involves matrix-Gla protein (MGP), which is the most potent inhibitor of arterial calcification which belongs to Gla-containing proteins also known as Gla-rich protein GRP [1,3,4,7,11,13,16]. GRPs are dependent on vitamin K which is a cofactor of conversion of glutamate into Gla [1,3,16]. MGP is a small protein with a molecular weight of 12 kDa, which contains 84 amino-acids, five glutamate (Glu), and three serine residues [16]. MGP is released from chondrocytes, arterial medial vascular smooth muscle cells (VSMCs), fibroblasts and endothelial cells [3,10,11,12]. MGP is widely found in soft tissues, especially in cartilages, blood vessels, arterial wall, heart, lungs, kidney and skin [3,10,11,12]. MGP inhibits arterial calcification via several pathways. MGP has a negative charge and therefore has a high affinity for free calcium. What is more, it can be directly related to circulating calcium molecules and hydroxyapatite crystals that are present in the vessel wall forming inactive complexes [16].

MGP is secreted in an inactive form and its activation by VK-dependent carboxylation is necessary for a potent calcification inhibitory effect [2,10]. Activated MGP binds calcium with a high affinity and inhibits the process of elastic fiber damage and VC [2,10]. It is worth noting that MGP is at present the only known factor which may reverse the process of VC [2]. The marked dependence was observed between a deficiency of VK and an elevation of uncarboxylated VK-dependent protein levels, as well as the hepatic protein induced by vitamin K absence-II (PIVKAII) and extrahepatic dephosphorylated-uncarboxylated matrix Gla protein (dp-ucMGP) [2]. It was found that VK influences the plasma level of dp-ucMGP, which decrease depending on the dose of VK taken [2]. The direct correlation was shown between circulating plasma dp-ucMGP levels and the severity of VC, cardiac function and long-term mortality [2]. An independent association was found between elevated plasma concentration of dp-ucMGP, lower levels of total uncarboxylated matrix Gla protein (t-ucMGP) and the severity of peripheral artery calcification in diabetic patients with high CV risk [2]. What is more, an independent relationship was found between higher dp-ucMGP values and carotid-femoral pulse wave velocity (cfPWV) in diabetic and CKD patients, which may lead to large arterial stiffening [2,17,18].

There are different types of MGP: t-ucMGP—the circulating variant which consists mainly of phosphorylated ucMGP (p-ucMGP), dephosphorylated-uncarboxylated MGP (dp-ucMGP), which is converting to dephosphorylated carboxylated MGP (dp-cMGP) [12]. It has been suggested that dp-ucMGP may be a predictor of peripheral arterial calcification independent from age, gender, previous CVD and t-ucMGP levels [12]. What is more, dp-ucMGP is related positively with peripheral artery calcification. Furthermore, the relationship was shown between high levels of dp-ucMGP and aortic calcification in patients at different CKD stages [12].

It was found that higher dietary intake of VK2, but not VK1, significantly reduced the incidences of VC and coronary heart disease (CHD) [19,20,21]. It may be because VK1 is mainly involved in carboxylation of VK-dependent factors in the liver, whereas VK2 takes part in carboxylation of VKDPs in the extrahepatic tissues [2,22]. The results of a recently performed 12-month prospective randomized trial indicate that VK1 may also participate in carboxylation reactions in extrahepatic tissues and can delay the progression of VC, however only when given in a high dose of 2 mg/d [23].

Recently obtained data indicate a close relationship between Gas 6 and the circulatory system [3]. Moreover, Gas 6 plasma level may be a prognostic factor of cardiovascular risk [3]. The activation of Akt and P13K by binding Gas 6 to Axl receptor limits the apoptosis of VSMCs [3]. Vitamin K_2_ can restore Gas 6 expression and activate downstream signaling by Axl, Akt and Bcl2 which can lead to inhibition of calcification and apoptosis of VSMC [3]. It was shown that Gas 6 is significantly released by VSMCs in human atherosclerotic plaques but not in healthy blood vessels [3]. The anti-inflammatory cytokine transforming growth factor β (TGF-β) induces the secretion of Gas 6 in VSMCs. On the other hand, Gas 6 stimulates VSMCs through suppressing the secretion of inflammatory factors, such as tumor necrosis factor (TNF) α and intracellular adhesion molecule (ICAM)-1 [3]. Therefore, Gas 6 can be considered as a protective factor in the atherosclerosis [3].

It is worth noticing that Gas 6 can stimulate endothelial progenitor cell (EPCs) proliferation and in vivo migration via activating the Akt signaling pathway [3]. EPCs play a key role in creating new blood vessels or in proliferation of pre-existing vasculature [3]. These data may create the basis for a further re-endothelialization therapy with the use of autologous EPC transplantation [3].

The first meta-analysis concerning dp-ucMGP as a risk factor for cardiovascular events and mortality was performed basing on the results obtained from 11 trials conducted on 33,289 patients [24]. The authors found that circulating dp-ucMGP was associated with an increased risk of all-cause mortality (HR 1.77; 95% CI 1.44–2.18; *p* = 0.476) and cardiovascular mortality (HR 1.84; 95% CI 1.33–2.55; *p* = 0.896) but not with the risk of CVD (HR 1.41; 95% CI 0.94–2.12; *p* = 0.068). Thus, higher dp-ucMGP levels led to a 70% increased risk for all-cause mortality as well as an 80% increased risk of CVD mortality. Nevertheless, the meta-analysis also demonstrated that the dietary intake of VK1 was not associated with the risk of all-cause mortality (HR 0.90; 95% CI 0.73–1.12; *p* = 0.030) and CVD mortality (HR 0.92; 95% CI 0.56–1.53; *p* = 0.146). Similarly, no significant associations between VK2 and the risk of all-cause mortality (HR 0.96; 95% CI 0.84–1.09; *p* = 0.707) and CVD mortality (HR 1.02; 95% CI 0.77–1.35; *p* = 0.644) were shown [24].

Another meta-analysis based on the data obtained from 21 articles, described clinical studies including 222,592 participants [25]. The authors found a significant relationship between VK1 intake and total CHD (pooled HR 0.92; 95% CI 0.84, 0.99; four studies), as well as VK2 and total CHD (0.70; 95% CI 0.53, 0.93; two studies). However, no significant associations were noted between a dietary consumption of VK and all-cause mortality, CVD mortality, or stroke. Elevated plasma dp-ucMGP was associated with an increased risk of all-cause mortality (1.84; 95% CI 1.48, 2.28; five studies) and CVD mortality (1.96; 95% CI 1.47, 2.61; two studies). The authors concluded that a higher dietary VK consumption was associated with a moderately lower risk of CHD, and higher plasma dp-ucMGP concentration was related to increased risks of all-cause and CVD mortality.

The results of the presented studies confirm the relationship between dietary VK consumption, plasma dp-ucMGP levels and calcification of blood vessels as well as cardiovascular risk. This indicates an important role of VK in these processes, which should be the subject of further research.

## 4. Vitamin K and Chronic Kidney Disease

Subclinical vitamin K deficiency often founds in CKD patients may be related to an increased risk of morbidity and mortality in this population [26]. The deficiency of vitamin K is characterized by low levels of circulating vitamin K and high inactive VKDP [3]. Low vitamin K intake and reduction in the carboxylation process of VKDPs are the main reasons of this status [3].

Several factors can influence VK status in CKD patients affecting VKDP activity [9]. Some of them lead to VK deficiency and cause decreased VKDPs activity such as: dietary restrictions associated with poor VK intake, dysbiosis due to the uremic condition, hemodialysis treatment, sevelamer (phosphate binder) or VKA (Vitamin K Antagonists). On the other hand, some factors can increase VKDPs activity: administration of calcimimetics, vitamin D (VD) analogs use, mycophenolate mofetil use and kidney transplantation [9]. Recently obtained data suggest that statin therapy may have an impact on VK metabolome [27]. A 41% reduction of MK-4 in kidney in mice fed with VK1 and concomitantly receiving atorvastatin was observed [22]. A clinical study conducted on hemodialysis patients treated with statins showed higher baseline coronary artery calcification (CAC) and greater progression of calcification [27].

It was reported that Gas 6 levels are increased both in CKD patients and in patients who underwent chronic hemodialysis [3]. The results of an animal study suggested that Gas 6 could protect from renal ischemia-reperfusion injury [28]. It was found that Gas 6 has beneficial impact on acute kidney injury due to its anti-inflammatory and immunoregulatory action [3]. It has been suggested that this effect may be related to endothelial activity as well as to the inflammatory process in these patients [3]. It was shown that disruption and inflammation of glomerular capillaries led to increase of Gas 6 levels [3]. Therefore Gas 6 is upregulated in many types of inflammatory nephropathy [3].

An experiment in a mouse model showed an increase in Gas 6 in acute kidney injury induced by sepsis with improved survival associated with decreased serum urea nitrogen, creatinine and renal tissue apoptosis [3]. Other investigators observed markedly reduced secretion of pro-inflammatory cytokines, such as interleukin (IL)-1β and TNF-α by Gas 6 action [3,28]. It was shown that Gas 6 (in its active form) inhibits vascular smooth muscle cells (VSMC) calcification via blocking apoptosis and preventing apoptotic VSMC vesicles to serve as nidi for calcium-phosphate precipitation [22]. Hyperphosphatemia-induced calcification of VSMC is related to downregulation of Gas 6 expression and VK2 inhibits VSMC calcification by restoring this Gas 6 anti-apoptotic pathway [22].

The results of clinical studies indicate a correlation between lower circulating ucMGP levels and a decrease in eGFR in patients with CHD [11,29]. However, albumin-to-creatinine ratio remained unaffected in this group of patients [11]. On the other hand, dp-ucMGP levels increased gradually with a decline in eGFR-estimated renal function in patients with varying degrees of renal dysfunction [17].

Dai et al. conducted a cohort study on 493 patients in the stage G5 of CKD (CKD G5) [30]. The authors assessed the relationship between functional vitamin K deficiency and all-cause mortality as well as whether this relationship was modified by the presence of VC in stage 5 CKD. Each increase in standard deviation in dp-ucMGP was associated with an increased risk of death from any cause (sub-hazard ratio (sHR) 1.17; 95% confidence interval, 1.01–1.37), adjusted to age, gender cardiovascular disease, diabetes mellitus, mass index, inflammation and dialysis treatment. This relationship was also significant after adjusting for CAC and AVC in the sub-analyzes (sHR 1.22, 1.01–1.48 and 1.27, 1.01–1.60, respectively). Thus, the authors showed that functional vitamin K deficiency was associated with an increased risk of death regardless of the presence of VC in patients with end-stage CKD [30].

Diabetic nephropathy is a complication commonly occurring in the patients with diabetes which often leads to end-stage renal disease (ESRD). Gas 6 seems to play a role also in this pathology. However, the obtained data on the role of Gas 6 in this nephropathy are controversial [3]. Thus, Nagai et al. showed an increased glomerular expression of Gas 6 and Axl in streptozocin-induced diabetic rats with glomerular hypertrophy [31]. On the contrary, Hung at al. in a study performed in the patients with type 2 diabetes observed not only an increase of Gas 6 plasma level but even a decrease of it [32]. Moreover, the value was inversely correlated with fasting blood glucose, tumor necrosis factor (TNF)-, interleukin (IL)-6, and vascular cell adhesion molecule (VCAM)-1 [32]. Other data suggested a decrease in plasma Gas 6 as proteinuria worsened [33]. It was suggested that the complex interaction between molecular charge and mass might play a role in glomerular filtration of Gas 6 [3,11]. Moreover, Gas 6 and albumin have similar molecular weight and charge and thus they may interact in the glomerular membrane [3,11]. Therefore, the plasma levels of Gas 6 may change in different stages of diabetes [3,11].

In diabetic patients with high CV risk an independent association between elevated levels of dp-ucMGP, lower levels of t-ucMGP and the severity of peripheral artery calcification was shown [2,34]. What is more, an independent relationship between higher dp-ucMGP values and cfPWV in diabetic and CKD patients was found, which can lead to stiffening of large arteries [2,17,18].

A recently conducted prospective study on 66 diabetic CKD patients showed that high plasma dp-ucMGP levels (≥656 pM) were associated with all-cause mortality (Hazard ratio-HR = 2.63, 95% CI = 1.17–5.94, *p* = 0.02), CV mortality (HR = 2.82, 95% CI = 1.07–7.49, *p* = 0.037) and progression of CKD (HR = 4.02, 95% CI = 1.20–13.46, *p* = 0.024) [35]. These data are in agreement with the results obtained from the prospective general population-based Prevention of Renal and Vascular End-Stage Disease (PREVEND) study which included 4275 participants [36]. Functional vitamin K deficiency (dp-ucMGP > 500 pmol/L) was detected in 31% of the entire study population, and the incidence was much higher among the elderly and those with comorbidities such as hypertension, type 2 diabetes, CKD and cardiovascular disease. The authors noted a significant relationship between high plasma dp-ucMGP levels with both all-cause (hazard ratio (HR) (95% confidence interval (CI)) = 0.20 (0.12–0.33), *p* < 0.001; squared term: 1.14 (1.10–1.17), *p* < 0.001) and cardiovascular mortality (linear term: 0.12 (0.05–0.27), *p* < 0.001; squared term: 1.17 (1.11–1.23), *p* < 0.001) [36].

Chronic kidney disease (CKD) predisposes to early vascular ageing (EVA) mediated by medial VC [37]. The obtained data indicate that cellular aging and inflammation caused by deoxyribonucleic acid (DNA) damage can lead to pathological conditions characterized by accelerated EVA. It was shown that nuclear factor erythroid 2–related factor 2 (NRF2) signaling and VK played a very important role in counteracting oxidative stress, DNA damage, senescence and inflammation. Thus, it is supposed that NRF2 activation and vitamin K supplementation may provide a novel treatment target for EVA [37].

In recent years, there has been a growing interest in the role of vitamin K in the calcification of arteries, especially in patients with CKD [1,2,3,4,5,7,8,11,12,13,16]. In patients with CKD, VC occurs widely even at early stages [1,2]. For example, CAC occurred in 13% of patients without renal disease, in 40% of non-dialyzed patients with CKD, in 57% of patients starting dialysis and in 83% of patients on long-term dialysis [1].

MGP plays an important role as an inhibitor of the calcium deposition and crystallization in the blood vessel wall [12]. MGP may slow the progression of vascular calcification in CKD patients by binding hydroxyapatite crystals [26]. This impairs their deposition and promotes macrophage-mediated clearance [26]. Moreover, the interaction between active MGP and bone morphogenetic protein-2 (BMP-2) leads to the inhibition of VSMC osteoblast transformation, which plays a pivotal role in the development of VC.

VK deficiency elevates the production of uncarboxylated MGP (ucMGP). Enhanced concentrations of ucMGP on arterial walls are associated with the severity of the VC [26]. Deposits present in the blood vessels may explain the low circulating levels of ucMGP, which are often observed in patients with ESRD on chronic hemodialysis [26]. As has been found in many studies, the concentration of circulating dp-ucMGP increases gradually in the subsequent stages of CKD [26]. Its levels have been associated with the severity of aortic calcifications (AC) and vascular stiffness (VS) [26]. It has been suggested that dp-ucMGP levels are associated with high-risk for cardiovascular (CV) mortality as well as all-cause mortality [2]. Moreover, dp-ucMGP levels are also associated with large artery stiffness [17].

Calciphylaxis, vascular calcifications, or calcifying uremic arteriolopathy (CUA) are rare life-threatening disorders, occurring mainly in patients with end-stage renal disease [38,39,40,41]. This disorder is characterized by calcification of cutaneous arterioles and rapidly progressive, very painful skin ulcerations [39]. Vascular calcifications are often observed in hemodialysis patients [38]. It was shown that the increased levels of dp-ucMGP observed in vitamin K-deficient patients may be associated with VC [8,38,39,40]. Higher plasma levels of uncarboxylated MGP (ucMGP) and carboxylated MGP (cMGP) were found in hemodialysis patients with calciphylaxis compared with patients receiving hemodialysis without calciphylaxis [42]. Vitamin K deficiency was associated with a lower concentration of cMGP and may be involved in the pathogenesis of calciphylaxis [42].

Mineral and bone defects are typical changes in patients with CKD which can develop even in the early stages of the disease [4]. Dietary potassium and phosphate limitation recommended for CKD are associated with a low consumption of green leafy foods, meat and dairy products rich in vitamin K. These dietary restrictions lead to VK deficiency often observed in these patients [4]. The expression of MGP in bone cells may be upregulated by VD [12]. The deficiency of VD is often observed in CKD patients [43]. It was shown that VD interacts with VKDPs. Preliminary studies showed that OC and MGP secretion rates increased after VD supplementation [43]. The active form of VD in osteoblasts increases the expression of osteocalcin associated with a vitamin K-dependent binding protein, which may support the mineralization of osteoblasts to osteocytes [43]. Moreover, VK is involved in osteogenesis of human mesenchymal stem cells by activation of VD_3_-mediated OC release [43]. The results of some clinical trials indicate that VD deficiency may have a synergistic effect in worsening the clinical consequences of VK deficiency [43]. Van Ballegooijen et al. investigated the association of both VD and VK status with all-cause mortality and death-censored graft failure in 461 kidney transplants recipients [44]. The authors found that low VD and VK levels were associated with increased mortality risk 2.33 (1.26–4.30) [HR (95% CI)] and increased graft failure risk 3.25 (1.17–9.08).

Poor VK status is often observed in patients with ESRD [45]. Evenepoel et al. performed an ancillary analysis based on data collected in the frame of prospective observational cohort studies assessing various aspects of bone health in de novo renal transplant recipients to investigate the association between VK status, inflammation, bone mineral density and clinical fracture incidents [44]. The authors found an association between VK deficiency and inflammation and low areal bone mineral density (aBMD) in ESRD patients which conferred an increased risk of incidents of fractures in this group of patients [44].

Hemodialysis patients often show a deficiency of vitamin K which is a strong predictor of vertebral fractures [1]. A typical diet for hemodialysis patients is usually poor in vitamin K [22]. Moreover, phosphate binders may have a negative effect on the bioavailability of vitamin K [22]. Alterations in the gut flora caused by drugs such as proton-pump inhibitors and antibiotics may also influence vitamin K bioavailability. Beta-lactam antibiotics with a N-methyl-thio-tetrazole side chain inhibit hepatic vitamin K epoxide reductase (VKOR), which is needed to convert vitamin K epoxide into reduced vitamin K [22]. Clinical observations conducted by Cranenburg et al. in 40 hemodialysis patients indicate a low intake of both VK1 and VK2 and elevated levels of non-carboxylated bone and coagulation proteins as well as very high levels of non-carboxylated matrix Gla proteins [46]. It is worth noting that both peritoneal dialysis (PD) and hemodialysis patients showed a similar degree of vitamin K depletion [26]. The results of a cross-sectional study performed on 21 PD patients indicated a significant proportion of patients with subclinical vitamin K deficiency, defined by low serum phylloquinone concentrations (<0.4 nmol/L) and elevated %ucOC (>20%) at 23.8% and 100%, respectively [26].

Wikstrøm et al. investigated possible causes of low vitamin K levels in hemodialysis patients, such as low intake, washout during dialysis, or decreased absorption capacity [47]. An additional aim of the study was to investigate whether the biomarker dp-ucMGP is affected in this group of patients. VK deficiency was found in all study participants. The authors showed that the low vitamin K status was not associated with the removal of VK during dialysis or decreased absorption but it was probably due to a low dietary intake of VK. It was also noted that dp-ucMGP was washed out during dialysis, however it was not affected by protein intake.

An observational study—VItamin K Italian (VIKI) Dialysis Study was carried out to estimate the prevalence of VK deficiency and to assess the relationship between VK status, vertebral fractures, VC, and survival in 387 hemodialysis patients for ≥1 year [48]. It was found that significant proportions of patients had deficiency of MK7 (35.4%), VK1 (23.5%), and MK4 (14.5%). Vertebral fractures were observed in 55.3% of patients, 80.6% had abdominal aorta calcification and 56.1% had iliac calcification. VK1 deficiency was found as the strongest predictor of vertebral fractures (odds ratio [OR], 2.94; 95% confidence interval [CI], 1.38–6.26), deficiency of MK4 was a predictor of aortic calcification (OR, 2.82; 95% CI, 1.14–7.01), while MK5 deficiency protected against it (OR, 0.38; 95% CI, 0.15–0.95). MK7 deficiency was a predictor of iliac calcification (OR, 1.64; 95% CI, 1.03–2.60). It was also noted that the presence of vertebral fractures was a predictor of VC (OR, 1.76; 95% CI, 1.00–3.08). The elevated levels of alkaline phosphatase and C-reactive protein (CRP), age and cerebrovascular events were considered as prognostic factors for mortality. The authors suggested that the VK system may play an important role in preserving bone mass and avoiding VC in hemodialysis patients [48].

Arteriovenous fistula (AVFs) is a typical vascular access type made for CKD patients requiring hemodialysis (HD) [7]. However, sometimes AVF failure may occur. There are two types of AVF failure. Early—caused by thrombosis or by an inability of the vein to dilate, and later—induced by stenosis and thrombosis resulting from neointimal hyperplasia (NIH) and calcification [7]. Calcification occurs in tunica media and intima in arterialized veins. Hemodialysis patients have generally poor VK status, and what is more, some of them receive VKAs which may promote calcification in vascular access, which is an independent predictor of mortality [7].

VK deficiency is often observed in CKD patients, both in the pre-dialysis period and in patients undergoing dialysis. Warfarin, when used in some CKD patients, may worsen this condition even more. Vitamin K deficiency promotes severe vascular calcification, especially arterial, which increases the risk of ischemic events. In addition, in this group of patients, mineral and bone effects are often observed, which may be intensified in the case of both VD and VK deficiency.

## 5. Vitamin K and Anticoagulant Therapy

Vitamin K levels in some CKD patients can be influenced by the anticoagulant warfarin administration [7,8,26,40]. This group of patients have a high risk of VC and calciphylaxis development associated with the use of vitamin K antagonists (VKAs) [4]. Warfarin is a selective inhibitor of oxidoreductase responsible for the regeneration of inactive vitamin K [26,49]. Different side effects may occur during warfarin treatment such as bone fractures and VC [7]. They may be caused by different mechanisms: direct—inhibition of carboxylation of OC and other bone matrix proteins, and indirect, resulting from limited an intake of foods rich in vitamin K [7].

The results obtained from animal models of CKD indicate, promoted by warfarin, calcium deposition in major blood vessels and arterial stiffening, however only in rats with kidney dysfunction [26]. These observations were confirmed by data obtained from clinical trials [26]. Some clinical data suggest that the risk of CUA in end-stage renal patients may by increased by warfarin treatment by a factor of 5 to 10 [39].

Hemodialysis patients receiving warfarin had an increased risk of developing severe AC and a long-term warfarin therapy was also associated with increased coronary and extra-coronary calcifications [26]. In a study performed in Japan warfarin was indicated as independent predictor of calciphylaxis, a severe complication of ESRD [26]. The results of a retrospective study included 41,425 hemodialysis patients showed that warfarin administration was associated with a higher mortality [26]. Additionally, an increased risk of stroke was also found in the subpopulation of patients with atrial fibrillation (AF) receiving warfarin. Thus, warfarin treatment in hemodialysis patients may not be beneficial [26].

Posch et al. [50] reported a more progressive reduction in estimated glomerular filtration rate in patients with AF in three and four stages of CKD receiving VKAs in comparison to the control group.

In contrast to these observations, results of the study conducted on pre-dialysis patients receiving VKAs indicate that the use of these anticoagulants was not associated with an accelerated kidney function decline or an earlier start of dialysis [51].

Novel oral anticoagulants (NOACs)—the new class of anticoagulant drugs are often used alternatively to VKAs [49]. Unlike VKAs NOACs do not interfere with the VK cycle [48]. Meta-analysis based on 12 randomized controlled trials (RCTs) showed that NOACs compared to warfarin significantly reduced the risk of any fracture by 18% (RR: 0.82, 95% CI: 0.73–0.93, *p* = 0.001) [52].

The impact of VK on VC progression in hemodialysis patients with AF receiving earlier VKAs, as well as after the withdrawal of these anticoagulants and replacement by rivaroxaban and supplementation with high dose of VK, was investigated in the Valkyrie Study [53]. It was found that withdrawal of VKAs and substitution by rivaroxaban with high-dose VK2 (2000 µg thrice weekly for 18 months) improved VK status in hemodialysis patients, but without any significant beneficial effect on VC progression.

In contrast, results of a cohort study conducted on 16,850 patients did not show significant differences in osteoporotic fracture occurrence in patients treated with warfarin compared to those receiving NOACs (dabigatran, apixaban, or rivaroxaban) [54]. Moreover, the rates of fractures were low in all compared groups. These findings are in agreement with the results of meta-analysis performed by Fioderlsi et al. who showed no significant increase in fracture risk with the use of VKAs versus NOACS [55].

The results of aforementioned studies and meta-analyses indicate a need for conducting RCTs to compare the occurrence of fracture risk between NOACs and VKA including CKD patients. Nevertheless, it should be taken into consideration that the use of NOACs in CKD patients is limited by the degree of renal function decline estimated by eGFR.

## 6. Vitamin K Supplementation

VK supplementation has been found to delay the progression of CAC and may delay the deterioration of arterial flexibility [1]. Vitamin K_2_ seems to be more effective at preventing and reversing arterial calcification compared to vitamin K_1_ [1,2]. Brandenburg et al. in the first randomized controlled trial conducted in patients with asymptomatic or mildly symptomatic aortic valve calcification (AVC) showed that VK supplementation may slow down the progression of this defect [23].

Some clinical data indicate that supplementation of VK2 may improve bone remodeling in hemodialysis patients with low serum parathyroid hormone (PTH) levels [1]. As it was mentioned above, CKD and hemodialysis patients often present vascular VK deficit because of low VK intake, which may result in higher risk of VC as well as bone fractures [2]. The results of some preliminary studies showed that both long-time (3 years) and medium-time (12 weeks) supplementation of MK-7 in different doses (100 µg/d, 180 µg/d and 360 µg/d) significantly decreased dp-ucMGP levels [2]. Busch et al. measured undercarboxylated and carboxylated osteocalcin levels as well as VK1 in 39 hemodialysis patients with renal osteodystrophy [56]. The authors found that hemodialysis patients had significantly higher levels of not only undercarboxylated osteocalcin, but also of carboxy-lated osteocalcin and thus total osteocalcin, which was probably associated with increased bone turnover. What is more, phylloquinone levels were also significantly higher in this group of patients, probably due to accumulation. It was worth noting that supplementation of VK1 had no impact on dp-ucMGP level in opposite to VK2 [41].

Kurnatowska et al. in one of the first interventional studies with VK2 supplementation in non-dialysed CKD patients showed a reduction in the progression of atherosclerosis with a significant decrease in the level of dp-ucMGP and total OC, however without a significant effect on the progression of vascular calcifications [57].

Results of clinical studies indicate that supplementation of VK lowers levels of dephosphorylated uncarboxylated MGP (dp-ucMGP) [4]. On the other hand, anti-vitamin K (AVK) therapy leads to increasing the levels of inactive dp-ucMGP and reducing dp-ucMGP by stopping treatment [4]. Therefore dp-ucMGP is considered to be a strong marker of VK status [4].

Meta-analysis performed by Cockayne et al. indicated that supplementation of VK2 significantly reduces hip (by 77%), vertebral (by 60%) and non-vertebral fractures (by 81%) [58]. Data obtained from some studies confirmed the relationship between vitamin K supplementation and lower risk of bones fracture [49,59].

A randomized, double-blind, parallel-group study was conducted to assess the impact of VK2 supplementation on arterial stiffness (AS) in patients in CKD [60]. The study included 159 patients with CKD stage 3b or 4 (eGFR15–45 mL/min per 1.73 m^2^). The parti-cipants were divided into two groups receiving orally 400 mg of VK2 (*n* = 80) or placebo (*n* = 79) once a day for 1 year. The authors showed that VK2 supplementation did not improve VS or other vascular measures.

The previously conducted study indicated that supplementation of hemodialysis patients with VK2 (menaquinone-7) resulted in a 61% reduction of dp-ucMGP levels [38]. Aoun et al. performed a prospective clinical study involving 50 hemodialysis patients [38]. The authors found that higher than 5000 pM levels of dp-ucMGP significantly correlated with VC scores (AC-24) and occurred more often in women, patients with recent fracture and patients with lower serum albumin. A daily dose of 360 μg of menaquinone-7 administered for 4 weeks, efficiently reduced dp-ucMGP levels in this population especially in diabetic patients.

Keyzer et al. conducted a long-time cohort study on 518 stable kidney transplant recipients to estimate association between VK depletion, defined as the levels of dp-ucMGP > 500 pmol/L, and all-cause mortality and transplant failure [61]. During a median follow-up of 9.8 (IQR, 8.5–10.2) years the authors found that the highest quartile of dp-ucMGP levels was related to a higher mortality risk (HR, 3.10; 95% CI, 1.87–5.12; *p* < 0.001) and developing of transplant failure (HR, 2.61; 95% CI, 1.22–5.57; *p* < 0.004). However, the last association was lost after adjustment for baseline kidney function (HR, 1.20; 95% CI, 0.52–2.75; *p* = 0.6).

On the other hand, results of the ViKTORIES trial performed in 90 patients after kidney transplantation with 1 year observation period showed no impact of VK supplementation on VS (treatment effect−0.23 [95% CI −0.75 to 0.29] × 10−3 mmHg−1; *p* = 0.377), VC (treatment effect−141 [95% CI −320 to 38] units; *p* = 0.124), nor any other outcome measure [62].

Results of animal studies clearly demonstrate that VK2 significantly improves matrix Gla protein carboxylation [4]. Arterialized human vein samples taken at calcification and neointimal hyperplasia sites did contain inactive matrix Gla protein, indicating local VK deficiency. It was shown that VKA has negative effects on AVF remodeling and VK2 decreased neointimal hyperplasia and calcification. Therefore, it has been suggested that supplementation of VK2 may reduce the risk of occurrence of neointimal hyperplasia and calcification in arterialized veins [4].

It is worth considering the recommendation of supplementing with VK in patients with high risk of CV events especially those with CKD and diabetes. The results of the studies conducted so far suggest that supplementation of VK even in high doses can be safe and beneficial in those patients. It was reported that VK2 supplementation may activate MGP in dialysis patients [35]. Moreover, it has been shown that VK2 supplementation in a dose-dependent manner decreased circulating uc-MGP plasma levels [39].

The randomized study was performed to estimate the effect of VK1 supplementation (10 mg after each hemodialysis for 1 year) on vascular calcification as well as to evaluate its impact on MGP in hemodialysis patients [63]. After one year of vitamin K supplementation the authors found a significant increase in MGP levels, however without any significant changes in carotid intimal medial thickness (CIMT) and abdominal aorta calcification score (AACS) compared to the baseline levels. On the other hand, the CIMT and AACS significantly increased in the control group (No-Vitamin K supplementation). The authors concluded that VK supplementation could not stop VC but significantly attenuated their progression [63].

A systematic review and meta-analysis were conducted to evaluate the impact of VK supplementation on VS and VC and relationship between inactive VKDPs levels and incident of cardiovascular disease and mortality [64]. The authors relied on results of 13 controlled clinical trials (*n* = 2162) and 14 longitudinal studies (*n* = 10,726). It has been demonstrated that VK supplementation was associated with significant reduction in VC (−9.1% (95% CI −17.7 to −0.5); *p* = 0.04) and VKDPs: dp-ucMGP (−44.7%; 95% CI −65.1 to −24.3, *p* < 0.0001) and ucOC (−12.0%; 95% CI −16.7 to −7.2, *p* < 0.0001) in comparison to control, with a non-significant improvement in VS. Longitudinal studies with median follow-up of 7.8 (IQR 4.9–11.3) years indicated the relationship of VKDP levels with a combined endpoint of CVD or mortality (HR 0.45 (95% CI 0.07 to 0.83), *p* = 0.02). Thus, the authors concluded that supplementation with VK significantly reduced VC, but not VS and VKDPs were associated with combined endpoint of CVD or mortality. However, these conclusions were limited by small numbers of studies with substantial heterogeneity [64].

Another meta-analysis based on 11 studies, involving 33,289 patients, was performed to assess the association between VK status and cardiovascular events or mortality [24]. The authors found the significant relationship between circulating dp-ucMGP and an increased risk of all-cause mortality (HR 1.77; 95% CI 1.44–2.18; *p* = 0.476) and cardiovascular mortality (HR 1.84; 95% CI 1.33–2.55; *p* = 0.896). However, dp-ucMGP was not associated with the risk of CVD (HR 1.41; 95% CI 0.94–2.12; *p* = 0.068). The meta-analysis also showed no relationship between dietary supplementation of VK1 or VK2 and the risk of all-cause mortality as well as CVD mortality [24].

The KING trial (vitamin K2 In reNal Graft) was a single-arm study performed to estimate the relationship between the changes in vitamin K status and indices of AS following 8 weeks of menaquinone-7 (VK2) supplementation (360 μg once daily) in 60 patients after renal transplantation [65]. Administration of VK2 caused a 14.2% reduction in mean cfPWV as well as 55.1% reduction of mean dp-ucMGP concentrations with a reduction in the prevalence of subclinical VK deficiency by 40%. The authors concluded that supplementation of VK2 was associated with improvement in subclinical VK deficiency and AS [65].

The results of clinical trials concerning vitamin K supplementation are presented in Table 1.

## 7. Conclusions and Comments

Vitamin K is proposed for a supplementary and protection treatment in patients with high risk of VC or bone disorders especially in CKD patients. The results obtained so far are not unequivocal. Nevertheless, as yet, there are no large RCTs conducted on CKD patients indicating vitamin K supplementation as preventive or nephroprotective therapy based on for the development of VC and the associated cardiovascular morbidity and mortality.

What is more, the question remains what kind of vitamin K should be supplemented in these patients—VK1 or VK2? It seems that VK2, which is very important for the extrahepatic VKDPs, could be probably harmless and relatively inexpensive [38]. However, there no adequately performed controlled trials confirming this conception. On the other hand, VK1 could be used alternatively because it is transformed into VK2 but in 10 times higher doses than for VK2 [38].

Another problem is that recommendations for a daily intake of VK differ depending on the country and source [9]. Nowadays, recommended intakes of VK1 range from 55 to 90 μg/d for healthy adult women and 65 to 120 μg/d for healthy adult men [4]. Daily doses of VK proposed by the Institute of Medicine in the United States are 120 μg for men and 90 μg for women [1,9]. On the other hand, the doses of VK recommended by The Italian LARN (Reference Levels of Assumption of nutrients and energy) are much higher and depending on age ranges: 140 μg/d for 18–59-year-olds and 170 μg/d for >60-year-olds [9]. In contrary to recommendations above, The Belgian Conseil Supérieur de la Santé recommended a daily intake of 50–70 μg/d of vitamin K_1_ for the adult population [9]. Thus, a consensus aimed at determining the adequate intake for VK does not exist [9].

Therefore, further randomized studies based on large populations should be carried out to answer the questions mentioned above.

## Figures and Tables

**Table 1:** Effects of supplementation of vitamin K—clinical trials.

Authors, Year, [Ref.]	Vitamin K Type, Dosage and Follow-Up	Type of Patients	Outcomes
Geleijnse et al. 2004 [19]	diet rich in VK1 mean intake of VK1: <200 µg/d, 200–278 µg/d and >278 µg/d diet rich in VK2 mean intake of VK1: <21.6 µg/d, 21.6–32.7 µg/d and >32.7 µg/d 7–10 years	Women and men aged ≥55 years without MI *n* = 4807	VK1—no association with incidents of CHD mortality, all-cause mortality and aortic calcification VK2—reduction of CHD mortality and inverse relation to all-cause mortality and severe aortic calcification
Gast et al. 2009 [20]	Mean VK1 intake 211.7 ± 100.3 µg/d Mean VK2 intake 29 ± 12.8 µg/d 8.1 ± 1.6 years	Postmenopausal women *n* = 16,057	Inverse association between VK2 intake and risk of CHD; no significant relationship for VK1 intake
Haugsgjerd et al. [21]	VK1 intake median 48 µg/d/1000 kcal VK2 intake median 7 µg/d/1000 kcal 11 years	Men and women aged 46–49 years *n* = 2987	No association between VK1 and CHD Higher intake of VK2 is related with lower risk of CHD (*p* = 0.03)
Brandenburg et al. 2017 [23]	VK1 2 mg/d *n* = 38 PL *n* = 34 for 12 months	patients with asymptomatic or mildly symptomatic AVC *n* = 72	Lower progression of AVC by 12% (*p* = 0.03) after VK1 vs. PL ↓ plasma dp-ucMGP by 45% (*p* < 0.001) in the VK1 group;
Kurnatowska et al. 2015 [57]	VK2 90 µg/d + Vit. D 10 µg/d *n* = 29 or Vit. D 10 µg/d alone *n* = 13 for 270 ± 12 days	non-dialyzed patients with CKD in stages 3–5 *n* = 42	VK2 + VitD—lower increase of CCA-IMT (*p* = 0.005) compared to VitD alone VK2 + VitD: ↓ dp-ucMGP (*p* = 0.02), OC (*p* = 0.04) and OPG (*p* = 0.02) levels
Witham et al. [60]	VK2 400 µg/d or PL for 1 year	patients with CKD in stages 3b or 4 *n* = 159	No effect on carotid-femoral PWV (primary outcome), AI, BP, B-type natriuretic peptide and physical function (secondary outcomes)
Aoun et al. 2017 [38]	VK2 (menaquinone-7) 360 μg/d for 4 weeks	hemodialysis adult patients *n* = 50	↓ dp-ucMGP plasma levels (*p* = 0.01)
Lees et al. 2021 [62]	VK3 (menadiol diphosphate) 5 mg/d *n* = 45 or PL *n* = 45 thrice weekly for 12 months	kidney transplant recipients *n* = 90	No impact on vascular stiffness and vascular calcifications
Mosa et al. 2020 [63]	VK1 10 mg after each dialysis for 1 year *n* = 20 or No VK1 *n* = 20	adult patients with ESRD regularly hemodialysed *n* = 40	↑ in MGP levels (*p* < 0.05) in VK1 group VK1—no significant changes in CIMT and AACS (no significant progression) No VK group—↑ CIMT (*p* < 0.005) and ↑ AACS (*p* < 0.005) (significant progression)
Mansour et al. 2017 [65]	VK2 360 μg/d for 8 weeks	renal transplant recipients *n* = 60	a 14.2% reduction in mean cfPWV (*p* < 0.001) ↓ dp-ucMGP by 55.1% with a ↓ in the prevalence of subclinical deficiency by 40% (*p* = 0.001) improvement in AS related independently with the ↓ dp-ucMGP (*p* = 0.014)

VK1—vitamin K_1_, VK2—vitamin K_2_, VK3—vitamin K_3_, MI—myocardial infarction, AVC—aortic valve calcification, PL—placebo, dp-ucMGP—dephosphorylated uncarboxylated MGP, CKD—chronic kidney disease, CCA-IMT—common carotid intima-media thickness, OC—osteocalcin, OPG—osteoprotegrin, PWV—pulse wave velocity, AI—augmentation index, BP—blood pressure, ESRD—end stage renal disease, CIMT—carotid intima media thickness, AACS—abdominal aorta calcification score, cfPWV—carotid-femoral pulse wave velocity.

## Data Availability

Not applicable.

## Abbreviations

AACS	abdominal aorta calcification score
AC	aortic calcifications
aBMD	areal bone mineral density
AF	atrial fibrillation
AS	arterial stiffness
AVC	aortic valve calcification
BMP-2	bone morphogenetic protein-2
CAC	coronary artery calcification
cfPWV	carotid-femoral pulse wave velocity
CHD	coronary heart disease
CI	Confidence Interval
CIMT	carotid intimal medial thickness
CKD cMGP	chronic kidney disease carboxylated matrix Gla protein
CUA	calcific uremic arteriolopathy
CVD	cardiovascular disease
dp-cMGP	dephosphorylated-carboxylated MGP
dp-ucMGP	dephosphorylated-uncarboxylated MGP
EPCs	endothelial progenitor cells
ESRD	end-stage renal disease
EVA	early vascular ageing
Gla Glu HR	γ-carboxyglutamate glutamate hazard ratio
MGP	Matrix Gla Protein
MK-4	menaquinone-4
NOACs	novel oral anticoagulants
NRF2	erythroid 2–related factor 2
OC	osteocalcin
PIVKAII	hepatic protein induced by vitamin K absence-II
PD	peritoneal dialysis
PTH	parathyroid hormone
p-ucMGP	phosphorylated ucMGP
RCTs	randomized controlled trials
sHR	sub-hazard ratio
t-ucMGP	total uncarboxylated Matrix Gla Protein
ucMGP	uncarboxylated Matrix Gla Protein
ucOC	undercarboxylated osteocalcin
VC	vascular calcification
VD	vitamin D
VK	vitamin K
VK1	vitamin K_1_
VK2	vitamin K_2_
VKA	Vitamin K Antagonist
VKDP(s)	VK-dependent protein(s)
VKOR	vitamin K epoxide reductase
VS	vascular stiffness
VSMC	vascular smooth muscle cells
